# Dystroglycan, Tks5 and Src Mediated Assembly of Podosomes in Myoblasts

**DOI:** 10.1371/journal.pone.0003638

**Published:** 2008-11-04

**Authors:** Oliver Thompson, Iivari Kleino, Luca Crimaldi, Mario Gimona, Kalle Saksela, Steve J. Winder

**Affiliations:** 1 Department of Biomedical Science, University of Sheffield, Western Bank, Sheffield, United Kingdom; 2 Department of Virology, Haartman Institute, University of Helsinki and Helsinki University Hospital, Helsinki, Finland; 3 Department of Cell Biology and Oncology, Consorzio Mario Negri Sud, Santa Maria Imbaro, Chieti, Italy; University of Edinburgh, United Kingdom

## Abstract

**Background:**

Dystroglycan is a ubiquitously expressed cell adhesion receptor best understood in its role as part of the dystrophin glycoprotein complex of mature skeletal muscle. Less is known of the role of dystroglycan in more fundamental aspects of cell adhesion in other cell types, nor of its role in myoblast cell adhesion.

**Principal Findings:**

We have examined the role of dystroglycan in the early stages of myoblast adhesion and spreading and found that dystroglycan initially associates with other adhesion proteins in large puncta morphologically similar to podosomes. Using a human SH3 domain phage display library we identified Tks5, a key regulator of podosomes, as interacting with β-dystroglycan. We verified the interaction by immunoprecipitation, GST-pulldown and immunfluorescence localisation. Both proteins localise to puncta during early phases of spreading, but importantly following stimulation with phorbol ester, also localise to structures indistinguishable from podosomes. Dystroglycan overexpression inhibited podosome formation by sequestering Tks5 and Src. Mutation of dystroglycan tyrosine 890, previously identified as a Src substrate, restored podosome formation.

**Conclusions:**

We propose therefore, that Src-dependent phosphorylation of β-dystroglycan results in the formation of a Src/dystroglycan complex that drives the SH3-mediated association between dystroglycan and Tks5 which together regulate podosome formation in myoblasts.

## Introduction

Cellular adhesion, migration and invasion are fundamental properties of most cell types during development and normal tissue/cellular function. For these cells, cell-cell and cell-substrate adhesion is essential to the maintenance of polarity, differentiation and tissue architecture. During development some cells, including myoblasts [1], migrate large distances. Others, such as macrophages are actively migratory as part of their normal function. In both instances, in order to migrate these cells need to overcome the physical and biochemical barrier of the extracellular matrix (ECM). Matrix degradation is most often seen at specialised adhesion sites known as podosomes or adhesion/protrusion sites known as invadopodia, reviewed in [2]. Such sites are proposed to mediate the polarised migration of cells that cross ECM boundaries. Podosomes are transient peripheral adhesion structures often formed in migrating tumour and other cells and contain a dense actin-rich core and a ring of actin regulatory/binding and adhesion proteins. Podosome assembly is regulated by non-receptor tyrosine kinases such as Src [3] and by Rho family GTPases [4], [5]. Related structures called invadopodia contain a similar complement of proteins, but are larger, centrally located and more persistent. In addition to which they have the ability to invade the matrix beneath them presumably as a prelude to tissue invasion [6]. We have demonstrated a key role for dystroglycan (DG) in cell migration and adhesion [7], [8] and recently identified DG as a component of podosomes.

Dystroglycan was first identified as a laminin binding protein from brain, and as part of the dystrophin glycoprotein complex (DGC) of skeletal muscle [9], [10]. DG is a transmembrane adhesion receptor comprising α- and β-subunits that are post-translationally cleaved from a single precursor peptide and subjected to extensive and functionally important glycosylation. The extracellular α-subunit mediates the link to laminin in the ECM and also binds the transmembrane β-subunit through non-covalent interactions, reviewed in [11]. As we have shown previously, the intracellular domain of β-DG mediates direct and indirect attachments to the actin cytoskeleton via a number of actin binding proteins, and also makes associations with a number of signalling and adaptor proteins (see [12] and references therein). Loss of DG or any of the associated DGC proteins in muscle leads to loss of the entire complex with loss of membrane-cytoskeleton stabilisation and consequent muscle damage [13]. DG is a key component of the DGC of skeletal muscle and plays an important role in costameric cell adhesion. By anchoring the costamere, the principle adhesion structure along the length of the individual myofibres, stably to the ECM, DG serves to preserve membrane integrity by protecting the cell against contraction induced membrane damage. In addition a number of signalling functions are also associated with DG [14]. In addition to a specific role in the maintenance of muscle integrity, DG has a more ubiquitous role in cell adhesion, signalling and polarity, and DG protein levels are reduced in almost all epithelial tumours so far examined [15]. We have identified DG as a component of small adhesion puncta during the early stages of myoblast spreading and characterised these puncta as podosomes containing a regulatory complex comprising dystroglycan, Tks5 and Src.

## Materials and Methods

### Cell culture

Mouse myoblast cells (H-2K^b^-tsA58) were maintained as described previously [16]. Myoblast cell lines that stably expressed dystroglycan-GFP, GFP, an shRNA against DG and a sense strand control shRNA were generated using retroviral infection as described previously [8], [12]. With the exception of the cytoplasmic domain of β-DG myc construct (cβDG-myc) all other dystroglycan expression constructs used including point mutants, whether fused to myc or GFP comprised full length α- and β-dystroglycan. A7r5 smooth muscle cells were grown in DMEM+10% FCS at 37°C in 5% CO_2_. Where required cells were plated onto glass coverslips coated overnight with 5 µg/ml in PBS of ECM protein; gelatin, laminin, fibronectin, poly-L-lysine (Sigma) or N-terminally-His-tagged E3 LG4-5 module of laminin α2 chain; [17] a generous gift from Erhard Hohenester (Imperial College, London). Uncoated regions were blocked with 1% BSA for 1 hour at room temperature, rinsed once with PBS and used within 3 hours of preparation. For matrix degradation assays, porcine type A gelatin (Sigma; 0.2% in 50 mM Na_2_B_4_O_7_ pH 9.3) was incubated with rhodamine B isothiocyanate (70 µM; Sigma) at room temperature for 2 hours. Following extensive dialysis, coverslips were coated in rhodamine-gelatin by immersion for 5 minutes. Excess was drained off and coverslips fixed in 0.5% (v/v) gluteraldehyde for 15 min. Coverslips were washed 3 times in PBS, incubated in ice-cold NaBH_4_ (3 min) with agitation, washed with PBS, and stored in 70% ethanol. Prior to use coverslips were dried and incubated in normal growth medium for 1 h. Podosomes/invadopodia were induced by addition of 2.5 µM phorbol 12, 13-dibutyrate (PDBu; Sigma) to the growth medium for 30 min (myoblasts) or 1 h (A7r5). Inhibition of Src was achieved by treatment in normal culture medium containing 10 µM PP2 (Calbiochem) for 1 hour prior to immunostaining. Tissue culture cells were fixed and stained for immunfluorescence microscopy as described previously [18]. Percentage of podosome containing cells, where quantified, were estimated in at least 100 cells in three independent experiments per treatment. The following antibodies were used for immunfluorescence microscopy at the indicated dilutions: MANDAG2 monoclonal anti-β-DG 1∶25 (1∶350 for western 1∶10 for IP) [19]; 1709, anti-pY892 β-DG 1∶10 [20]; Vinculin, clone V9131 1∶400 and Talin, clone 8D4 1∶100 (Sigma-Aldrich); Phosphotyrosine PY20 1∶1000 and Paxillin 1∶1000 (Transduction Labs); Cortactin, clone 4F11 1∶100 (Upstate Biotech); Tks5 1∶200 (1∶20 for IP) and Myc clone 9E10 1∶100 (Santa Cruz Biotech); Tks5 1∶500 (1∶2000 for western) [21]; Ezrin 1∶1000 (Dr Sam Crouch, University of Dundee); Plectin clone 9F10 1∶10 000 (Gerhard Wiche, Vienna); Actin-related protein 1∶500 (Laura Machesky, CRUK Glasgow); Pan-Src 1∶40 (Calbiochem); Active Src (dual-phosphorylated) 1∶1000 (Cell Signalling); Glutathione-S-transferase 1∶5000 for western (SJ Winder unpublished); fluorescent phalloidins to detect F-actin 1 µg/ml (Molecular Probes) and fluorescent species specific secondary antibodies 1 µg/ml (Vector Labs).

For live cell imaging, myoblast cells plated on glass bottom culture dishes were transiently transfected as described above with GFP-actin [22] and imaged at 30 second intervals on either a Leica DMIRE2 using 63× lwd optics. Images were captured by CCD and converted to Quicktime movies by importing individual frames into ImageJ. Time lapse recording was also performed on a DeltaVision RT using 63× oil immersion optics. Movies were generated directly using the SoftWorx package.

### Assays for protein-protein interaction

Cytoplasmic domain of mouse β-DG expressed in E. coli and purified as described previously [23], was used to screen a phage display library comprising an essentially complete collection (n = 296) of human SH3 domains using the protocol described in Kärkkäinen et al. [24]. In short, recombinant β-DG was adhered to 6-well plastic plates (1 µg/ml in PBS incubated overnight at 4°C) and blocked with 5% (w/v) milk in PBS/0.05% (v/v) Tween 20. The wells washed briefly with PBS before 10^10^ pfu of infectious SH3 phage library was added per well. After 2 hours of incubation the wells were washed four times with PBS/0.05% Tween 20 and once with PBS followed by addition of log phase E. coli TG1 cells to be infected during 1 h incubation. A fraction of the infected cells were plated on ampicillin plates, and the rest of them put into culture and infected with M13KO7 helper phage (5×10^8^ pfu/ml) to generate a secondary infectious SH3 phage library. After selection of β-dystroglycan-binding phages as above the SH3 domains in the ampicillin resistant colonies obtained were identified by sequencing.

GST-fusion proteins corresponding to the 3^rd^ and 5^th^ SH3 domains of Tks5 were generated by PCR, expressed in E coli and purified on glutathione Sepharose according to the manufacturers instructions (GE Healthcare). To evaluate binding of β-DG to the GST-SH3 domains, myoblast lysate prepared in radio immune-precipitation assay (RIPA) buffer was passed over a GST-SH3 domain affinity column. Following extensive washing bound DG was eluted with the GST-SH3 domain using 20 mM glutathione. Fractions were separated by SDS-PAGE and analysed by western blotting as described previously [7]. Immunoprecipitation assays were carried out on myoblast extracts in RIPA buffer at 4°C overnight, following extensive washes and a final stringent wash in RIPA buffer containing 0.6 M LiCl_2_, bound proteins were analysed by SDS-PAGE and western blotting as above.

Cell fractionation experiments were performed as described previously (Spence et al., 2004). Briefly cells were disrupted in a 1% CHAPS buffer to release membrane associated and cytosolic proteins. Following centrifugation the resultant pellet was further extracted with 1% Triton and centrifuged to form a Triton-insoluble cytoskeletal pellet fraction, and a residual Triton soluble fraction.

### GFP-Tks5-mito construct

The GFP-Tks5-mito construct was generated by a two-step process. GFP was fused directly onto the N-terminus of Tks5, and an additional linker was added to the C-terminus of Tks5 separating Tks5 and the mito sequence. As first step, the GFP-mito vector was generated by cloning the cDNA of the mitochondrial targeting sequence of ActA [25] into pEGFP-C2 (Clontech), similarly to a prior strategy [26]. The additional linker GSTSGSGKPGSGEGSTKG [27] was inserted into the EcoRI site of the GFP-mito vector, between the GFP and the mito sequence. Finally, the human cDNA of Tks5 (generous gift from S. Courtneidge, La Jolla) was amplified by PCR using the following primers: FWD: 5′-ATG CTC GAG AAT GCT CGC CTA CTG CGT GCA-3′ and REV: 5′-AGG AAG CTT CCG TTC TTT TTC TCA AGG TAG TTG-3′, and cloned into the GFP-mito vector via XhoI-HindIII restriction sites.

## Results

### Dystroglycan in adhesion puncta and podosomes

**Figure 1:**
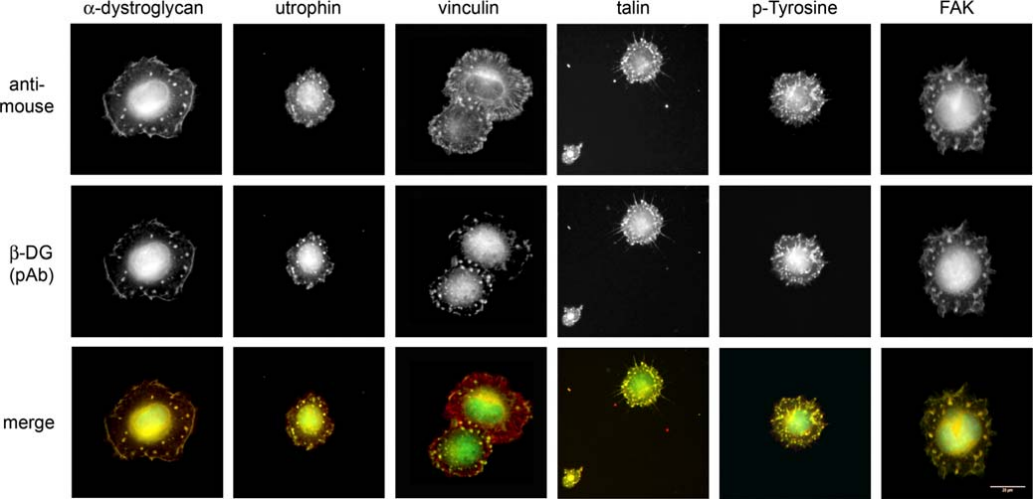
Spreading myoblasts form DG rich adhesion puncta. Myoblast cells were seeded onto tissue culture plastic and allowed to spread for one hour before fixation and staining for β-dystroglycan, and counterstained with one of the indicated cell adhesion proteins/markers. In all cases β-DG was colocalised in dense puncta either peripheral or more central to the cell, reminiscent of podosomes. Both α-dystroglycan and utrophin, components of a recognised adhesion structure, co-localised with β-dystroglycan. Other markers of classical focal adhesions such as vinculin, talin, FAK and the generic marker phospho-tyrosine also marked the β-dystroglycan containing puncta. Vinculin also clearly labelled peripheral focal adhesions, distinguishing them from the β-dystroglycan puncta. Scale bars 20 µm.

**Figure 2:**
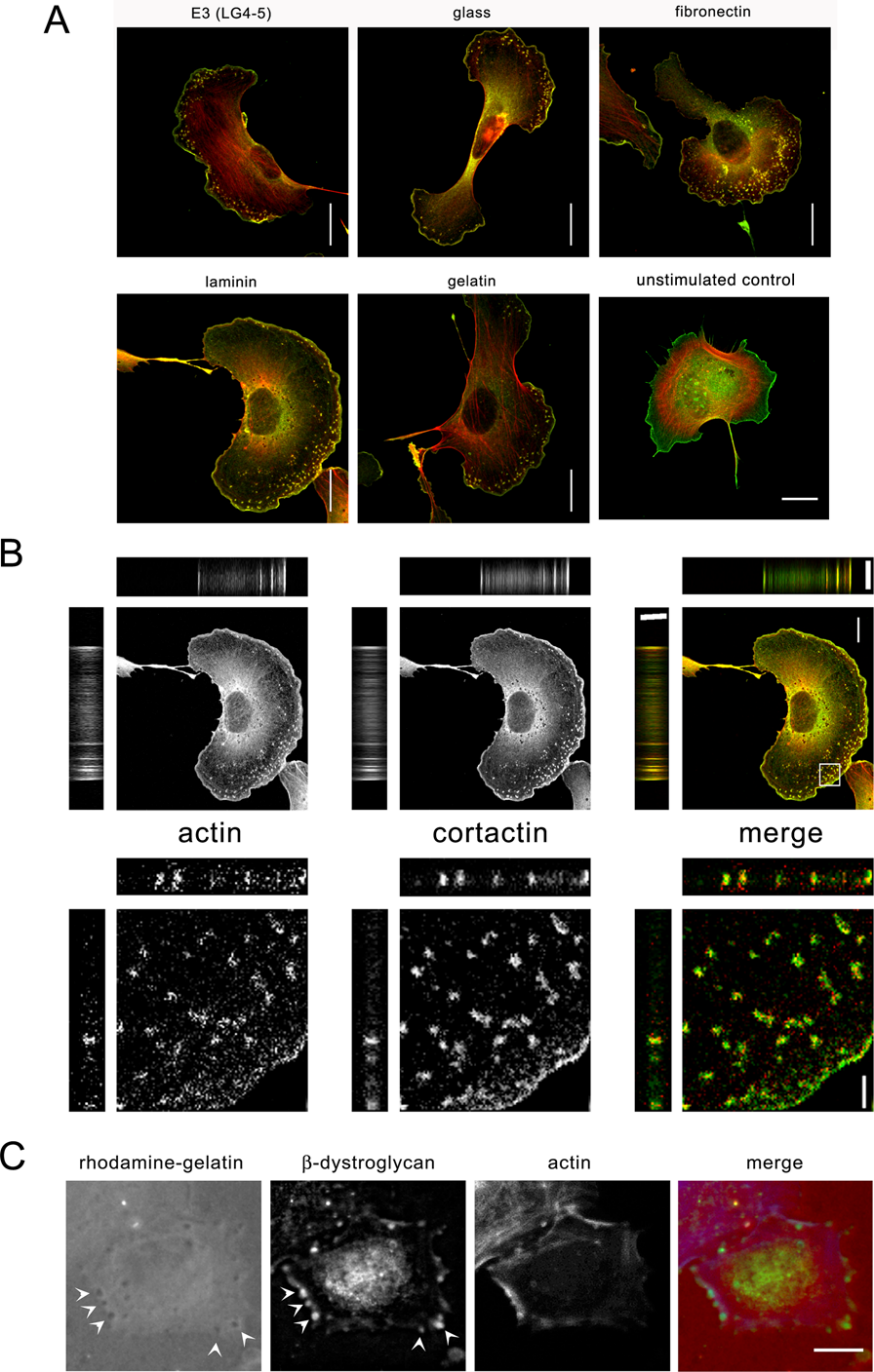
Myoblasts form podosomes in response to PDBu. To determine if myoblast cells could form podosomes, myoblast cells were allowed to adhere and spread on various substrates, and stimulated with PDBu for 30 min and fixed and stained for the podosome markers cortactin (green) and F-actin (red). Myoblasts formed peripheral actin and cortactin containing puncta on all substrates tested. An unstimulated cell grown on a glass coverslip is also shown for comparison (A, bottom right panel). These structures are morphologically indistinguishable from podosomes (A). The structures appeared columnar within the cell and cortactin was localised around a more central actin core as seen in the Z section of the zoomed region of B. The identity of the DG and actin-rich puncta as podosomes/invadopodia was further substantiated by the degradation of rhodamine-gelatin beneath them, seen as darker areas of reduced rhodamine gelatin (red in merge, arrowed) that are coincident with DG localisation (green in merge, arrowed) and F-actin (blue in merge). Scale bars 20 µm in A and 10 µm in B, C, 2 µm in zoomed region of B.

Whilst much is known about the role of dystroglycan in mature differentiated skeletal muscle [28], less is known about the functions of DG in cell adhesion in developing muscle [1]. We therefore examined the role of DG in the formation of cell adhesions during spreading and adhesion of myoblasts. During the early phases of cell spreading, and independent of the substrate on which the cells were spreading on, DG could be seen in relatively large and densely staining puncta (Figure 1). These puncta were often located more centrally, and appeared morphologically distinct from peripheral focal adhesion-like structures. Whilst both α- and β-dystroglycan as well as utrophin co-localised in these puncta, other more traditional adhesion proteins including vinculin, talin and FAK as well as the more generic phospho-tyrosine also typically seen in adhesion structures were also localised in the puncta (Figure 1). Interestingly, neither α- and β-dystroglycan nor utrophin appeared to localise in more peripheral focal adhesion type structures, compare vinculin staining in Figure 1. The dystroglycan-staining puncta do not appear morphologically to resemble focal adhesions and the differential staining of dystroglycan also suggests that the puncta are somehow different to focal adhesions stained by vinculin. Whilst it is possible that the puncta are focal contacts [29], [30] their more central position in the cell and their relatively large size would argue against this. One other adhesion structure that has a larger and rounder morphology is the podosome, typically found in cells of the monocyte lineage, osteoclasts and smooth muscle cells [6]. Myoblasts have not previously been demonstrated to form podosomes. Therefore in order to determine if myoblasts could form podosomes, we subjected well-spread myoblasts to stimulation with the phorbol ester PDBu, in order to activate Src downstream of PKC as part of a pathway leading to podosome formation [31]. Myoblast cells were then co-stained for classical markers of podosomes: actin and cortactin (Figure 2A). Podosomes contain a dense actin-rich core surrounded by a loose meshwork of adhesion proteins and actin binding and regulatory proteins [6]. In this regard the structures formed in myoblast cells appear morphologically similar to podosomes seen in other cell types [2] (Figure 2B) and thus henceforth will be considered as podosomes. Podosome formation was also independent of the substrate that the cells were adhered to. Actin- and cortactin-containing structures formed in a band behind the leading edge of the cell equally well on glass, gelatin, fibronectin and whole or E3 fragment of laminin, compared to the unstimulated cell (Figure 2A). A further characteristic of podosomes and structurally related invadopodia, is the ability to degrade the matrix beneath the adhesion site [6]. The dystroglycan-containing structures in myoblasts appear to share this property too, in that there are zones of clearance of rhodamine-gelatin beneath the dystroglycan-containing adhesion structures (Figure 2C). In addition, DG co-localised in podosomes with plectin and integrin, other proteins known to be resident in podosomes [6], [32] (Figure S1A). Other well-characterised myoblast cells such as C2C12 [33] were also able to form podosomes in response to PDBu (Figure S1B). To ascertain whether or not the localisation of DG to podosomes was unique to myoblasts we also examined the localisation of DG in A7r5 smooth muscle cells in response to PDBu stimulation. A7r5 cells are a well-established model for podosomes and as shown in Figure S1C, DG also colocalised in podosomes with actin, cortactin or vinculin. Thus it appears that myoblasts can form podosomes and that dystroglycan is localised to podosomes along with other well recognised podosome proteins. Furthermore as can be seen from movies of myoblast cells expressing GFP-β-actin (Movie S1, S2, S3), podosome structures and podosome dynamics appear qualitatively similar to GFP-β-actin movies from A7r5 cells for example see [34] and have similar dynamics to DsRed-SM22 (used to label F-actin) podosomes in A7r5 cells [35], [36].

### Dystroglycan interacts with Tks5

Given the apparent localisation of dystroglycan to podosomes (Figure 1 and Supp Figure 1) but not to focal adhesions (Figure 1) we reasoned that a unique factor may be targeting dystroglycan to podosomes. The DG cytoplasmic domain comprises 25% proline residues which form six PxxP motifs suggestive of potential SH3 domain interaction sites. Given the importance of SH3 domain adaptors in mediating connections between proteins at adhesion sites [37] we undertook a human proteome-wide SH3 domain phage-display screen using the cytoplasmic domain of β-DG as an affinity ligand, in order to identify potential adaptor proteins. In agreement with the predicted SH3 binding sites, significant enrichment of clones from the SH3 domain displaying phage library was observed upon selection with β-DG, some of these currently form the basis of ongoing studies in our laboratories. Sequencing of the clones identified the third SH3 domains of the homologous adapter proteins Tks5 and Tks4 [38] among the most frequent β-DG binders. Although the adapter protein Grb2 has been suggested in the literature as an SH3-containing binding partner of β-dystroglycan [39], neither of the two SH3 domains of Grb2 were found among the affinity selected clones. When we produced homogeneous phage preparations carrying either the third SH3 domain of Tks5, or the N- or C-terminal SH3 of Grb2, only Tks5 but not Grb2 SH3-displaying phages bound to β-dystroglycan-immobilized plates better than to negative control plates (data not shown). Moreover, no β-dystroglycan binding could be observed by Grb2 SH3 domains expressed as recombinant GST fusion proteins (data not shown). Thus, the failure to identify Grb2 SH3 domains in the phage library screen was in agreement with undetectable β-dystroglycan binding capacity in these individual assays, suggesting that the previous studies on Grb2 binding to β-dystroglycan [40] may have overestimated the affinities of these SH3 interactions. Based on use of this library for affinity selection of a large number of ligands, some with no known partners and some with well characterized SH3 partners, it has become clear that only moderately strong binding (with reference to SH3 binding in general) is needed in order to positively select individual SH3 clones from this library. Based on Kd values previously established for SH3 interactions that we have selected or failed to select in our screens, we have estimated that a binding affinity of approximately 10–20 µM would be generally required to support positive selection. Considering that selection of the third SH3 of Tks4 and Tks5 was relatively inefficient, we would anticipate the affinity in these cases to be in the 5–10 µM range. Compared to some other protein interactions, this would be rather weak, but among SH3-mediated interactions represents a perfectly respectable value.

Tks5 is a 140 kDa protein comprising an amino terminal PX domain followed by five SH3 domains and was first identified in a screen for Src substrates [38]. Importantly Tks5 has also been found to associate with ADAMs family proteases and to localise to podosomes in response to Src activation [21], [41]. Tks4 was identified from databases and like Tks5 has a PX domain but lacks the fourth of the five SH3 domains. More recently it has been suggested that Tks5 may undergo alternative splicing to remove the first SH3 domain [42], however the relationship between this putative alternatively spliced Tks5 and Tks4 are not clear. No further evidence of a role for Tks4 is available, we therefore concentrated our efforts on the Tks5 SH3 domain interaction.

**Figure 3:**
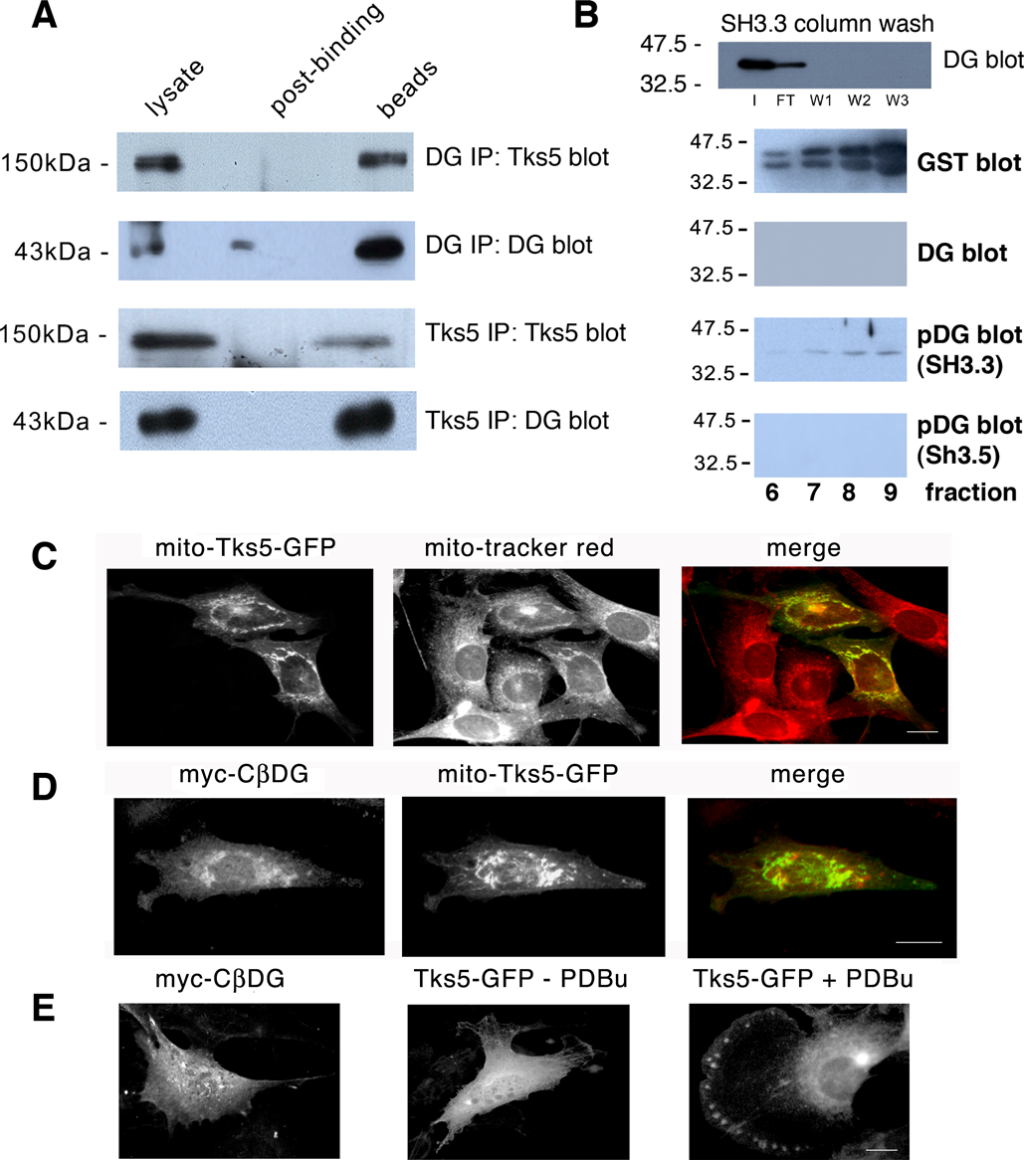
Interaction of DG with Tks5. A. β-DG (43 kDa) was co-immunoprecipitated from myoblast extracts with Tks5 antiserum and Tks5 (150 kDa) was co-immunoprecipitated with β-DG antibodies. Affinity purification of β-DG from myoblast extracts using the 3^rd^ and 5^th^ SH3 domains of Tks5 fused to GST revealed a specific interaction with only the 3^rd^ SH3 domain (B). Western blots show peak fractions eluting from the GST-SH3 column, furthermore the β-DG recovered from the GST-SH3 column was preferentially recognised by antibodies that recognise the tyrosine phosphorylated form of β-DG (pDG) and not the non-phosphorylated form (DG). A GFP-tagged Tks5 construct (mito-Tks5-GFP; C) that was targeted to the mitochondria in myoblast cells, was also able to recruit a myc-tagged β-DG cytoplasmic domain (myc-CβDG) to the mitochondria (D) indicating an association between the two proteins in a cellular context. Both myc-CβDG is diffusely distributed when expressed alone as is Tks5-GFP in the absence of PDBu, but in the presence of PDBu clearly targets to podosome structures (E). Scale bars 10 µm.

**Figure 4:**
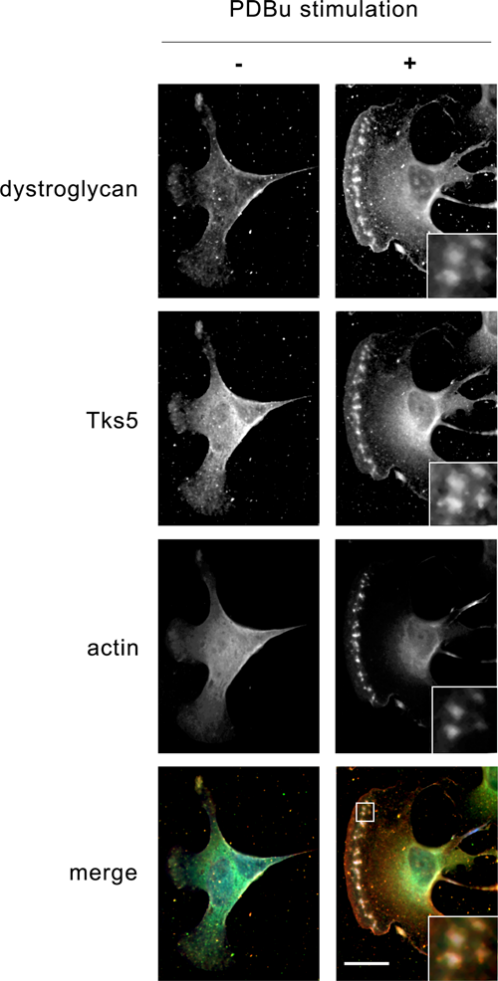
DG and Tks5 localise to podosomes in myoblasts. Myoblast cells, untreated (−) and following stimulation with PDBu for 30 min (+) were stained for endogenous β-DG (red), Tks5 (green) and F-actin (blue). Unstimulated cells exhibit only faint β-DG and Tks5 localisation toward the periphery in cellular protrusions, potentially in podosome cluster precursors [43]. Following stimulation, β-DG and Tks5 were clearly localised along with F-actin to podosome structures following stimulation. The actin staining can be seen clearly to form a more central core to the podosome with Tks5 and dystroglycan around the periphery (inset). Scale bar 20 µm.

We verified an interaction between Tks5 and β-DG by reciprocal immunoprecipitation from myoblast lysates. As shown in figure 3A, antibodies against either β-DG or Tks5 were able to immunoprecipitate the other protein. To further substantiate a role for the 3^rd^ SH3 domain we used a GST-SH3 pulldown to recover β-DG from myoblast lysates. DG was recovered specifically with the 3^rd^ but not the 5^th^ SH3 domain of Tks5 fused to GST. Significantly the β-DG bound to the Tks5 was only recognised by an anti-phospho β-DG antibody that is able to recognise DG that is phosphorylated on tyrosine 890 (Y890). A monoclonal antibody that recognises a similar epitope, but that can't bind to β-DG that is phosphorylated on Y890 did not detect any β-DG in either pulldown (Figure 3B). This result suggests that not only is Src activation necessary for podosome formation, and for the recruitment of Tks5 to podosomes, but that when β-DG is associated with Tks5 in cells, it too is phosphorylated on tyrosine at Y890. In order to demonstrate an interaction between DG and Tks5 in a cellular context, we employed a GFP-tagged Tks5 construct fused with a mitochondrial targeting sequence at its amino terminus. As can be seen in Figure 3C, the mito-Tks5-GFP construct was efficiently targeted to mitochondria and localised extensively with mito-tracker red staining in the myoblast cells. We then co-expressed the mito-Tks5-GFP construct with a construct comprising the cytoplasmic domain of β-DG only with an amino-terminal Myc tag. As we have demonstrated previously this construct is entirely cytosolic [8], [12], and therefore its localisation should respond to that of a binding partner that was localised to a discrete domain such as the mitochondrion. As can be seen, this is indeed the case; the cytoplasmic Myc-cβ-DG construct is colocalised with the mito-Tks5-GFP (Figure 3D) rather than appearing diffusely in the cytoplasm (Figure 3E) substantiating an interaction between the two proteins. We further verified the association between Tks5 and DG by immunfluorescence localisation of the two proteins with F-actin in myoblasts before and after stimulation with PDBu. As can be seen in Figure 4, whilst there is a faint and indistinct area of colocalisation between Tks5 and β-DG towards the leading extremities of unstimulated myoblasts, which may correspond to the podosome cluster precursors described by Matsudaira and colleagues [43], following PDBu stimulation all three proteins colocalise robustly to distinct podosome structures behind the leading edge of the cell. And as in typical podosome structures, there is a clear actin core with a more diffuse and wider distribution of dystroglycan and Tks5 (Figure 4 inset).

### Altering dystroglycan expression levels modulates podosome assembly

**Figure 5:**
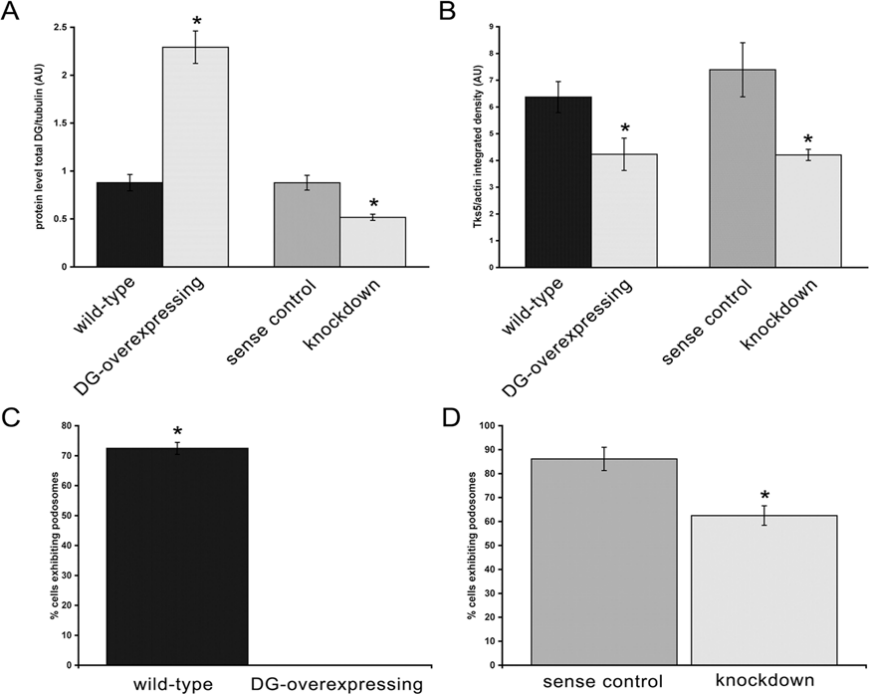
DG over-expression abolishes podosome formation. Quantification of dystroglycan and Tks5 protein levels in control myoblast cells (wild-type), cells stably expressing GFP-tagged full-length DG (DG-GFP), shRNA against DG (knockdown) or a control shRNA (sense). Dystroglycan levels for the four cell lines are shown in A, with corresponding Tks5 levels in B. Whilst dystroglycan levels were increased by 2.3-flod and decreased by half in overexpressing and knockdown cells respectively, Tks5 levels were reduced by approximately 33% in both cases. Wild-type, sense, knockdown and myoblasts transiently expressing a myc-tagged DG construct were induced to form podosomes with PDBu and the number of cells exhibiting podosomes counted following fixation and staining. DG depletion caused a modest reduction in cells with podosomes compared to control, whereas DG over-expression completely abolished podosome formation. (C,D; mean±SEM).

**Figure 6:**
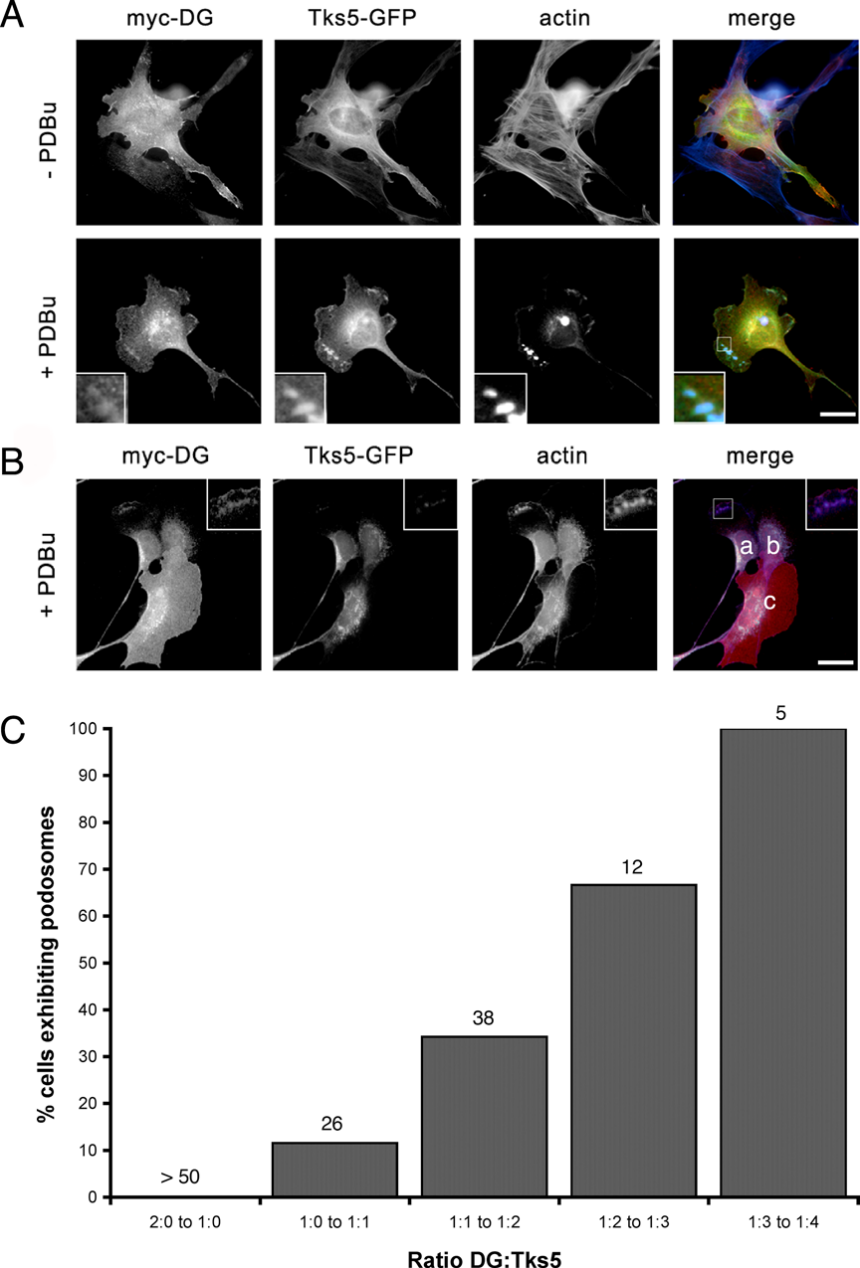
The dystroglycan/Tks5 ratio is important for podosome formation. Wild-type myoblast cells were co-transfected with myc-DG and Tks5-GFP and induced to form podosomes by stimulation with PDBu. In cells co-expressing both myc-DG and Tks5-GFP, podosomes were only formed when Tks5 expression was high, as judged by myc staining relative to GFP fluorescence; relative fluorescence ratio DG∶Tks5 1∶0.59 (A lower panels). However when myc-DG staining (red) predominated over GFP, podosomes were not formed in response to PDBU. B. cells marked in the merged image have DG∶Tks5 ratios of a. 1∶0.6; b, 1∶0.47; c, 1∶0.2. Cells are also stained for F-actin (blue). Scale bars are 10 µm. Quantitative analysis of dystroglycan-myc and Tks5-GFP ratios in transiently transfected cells reveal a ratio of Tks5∶DG that is permissive for podosome formation (C). Numbers above each column refer to numbers of cells falling within the given ratio of DG∶Tks5.

If as proposed Tks5 is a ‘master regulator’ of podosome assembly [21], and DG also associates with Tks5, DG may also have a regulatory role in the podosome assembly process. In order to address this we examined myoblast cells either overexpressing or depleted for DG (Figure 5A–C). As determined by assessing the characteristic actin morphology of podosomes and compared to control cells, siRNA-mediated depletion of DG to 50% of endogenous levels in myoblast cells had a modest but statistically significant effect on the number of cells producing podosomes in response to PDBu. By contrast, over-expression of DG (tagged with either Myc or GFP) dramatically and completely abolished the ability of myoblasts to form podosomes following PDBu stimulation (Figure 5D). In both dystroglycan knockdown and dystroglycan overexpressing cells there was a reduction in Tks5 to an approximately equal level. But despite this reduction in Tks5, the levels of reduction did not correlate with the change in podosome levels in these cells. Thus the effect on podosome assembly of altering DG expression levels either up or down was not a simple consequence of affecting Tks5 levels in the cell (Figure 5B,C) as Tks5 levels were equivalent in both knockdown and overexpressing cells. We hypothesised therefore, that in over-expressing DG we are potentially titrating out factors such as Tks5 that are crucial to podosome formation. The relative levels of Tks5 and dystroglycan would therefore appear to be as important in the regulation of podosome assembly as the actual levels of each protein. From this one could propose that podosome formation should be restored in cells overexpressing DG by the concomitant overexpression of Tks5 in the same cell, restoring the ratio of dystroglycan to Tks5. By co-transfecting myoblasts with Tks5-GFP and DG-Myc we could observe a restoration of podosome formation in response to PDBu, but only once Tks5 overexpression reached some threshold level of expression that balanced the level of DG-Myc overexpression and permitted podosome formation (Figure 6A). However, if DG-myc levels predominated over Tks5-GFP, podosome assembly is inhibited (Figure 6B). Quantification of the relative fluorescence intensity for the two fluors (GFP for Tks5 and rhodamine for DG-Myc) per µm^2^ of cell area in the cells in Figure 6A,B revealed that a fluorescence ratio of DG∶Tks5 of 1∶0.6 was permissive for podosome formation, whereas a DG∶Tks5 fluorescence ratios of 1∶0.47 or 1∶0.2 was inhibitory to podosome formation. These ratios represent ratios of fluorescence and not ratios of the actual proteins, but nonetheless changing the amount of dystroglycan relative to Tks5 appears to alter the ability of cells to form podosomes in a ‘dose-dependent’ manner (Fig. 6C). These data suggest the existence of a finely tuned regulatory balance between dystroglycan and Tks5 in podosome assembly.

### Src phosphorylation of dystroglycan is required for podosome formation

**Figure 7:**
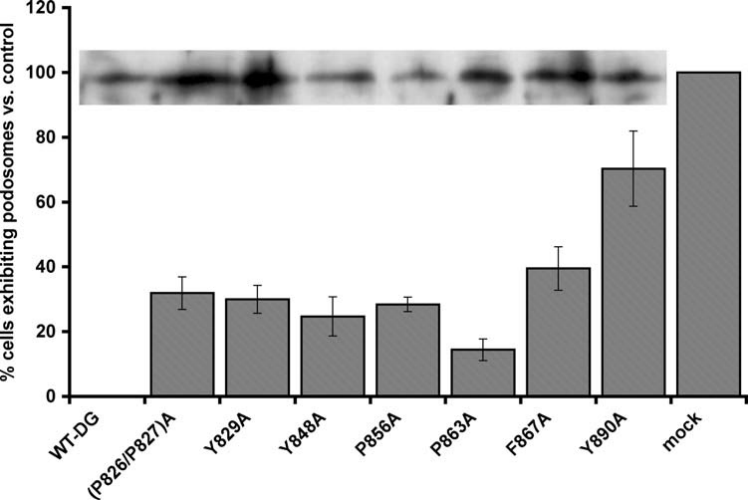
Mutation of the DG phosphorylation site restores podosome formation. Myoblast cells were transiently transfected with dystroglycan-GFP wild-type sequence, or containing the indicated point mutation in the cytoplasmic domain. Wild-type dystroglycan fully suppressed podosome formation, whereas all mutant constructs partially suppressed the inhibition of podosomes by DG over-expression by around 20–30%, the Y890A mutation however, suppressed the inhibitory effect by over 70% compared to mock transfected controls. (mean±SEM). Inset shows typical expression levels of the dystroglycan-GFP and mutants as judged by anti-GFP western blot from a representative transient expression.

**Figure 8:**
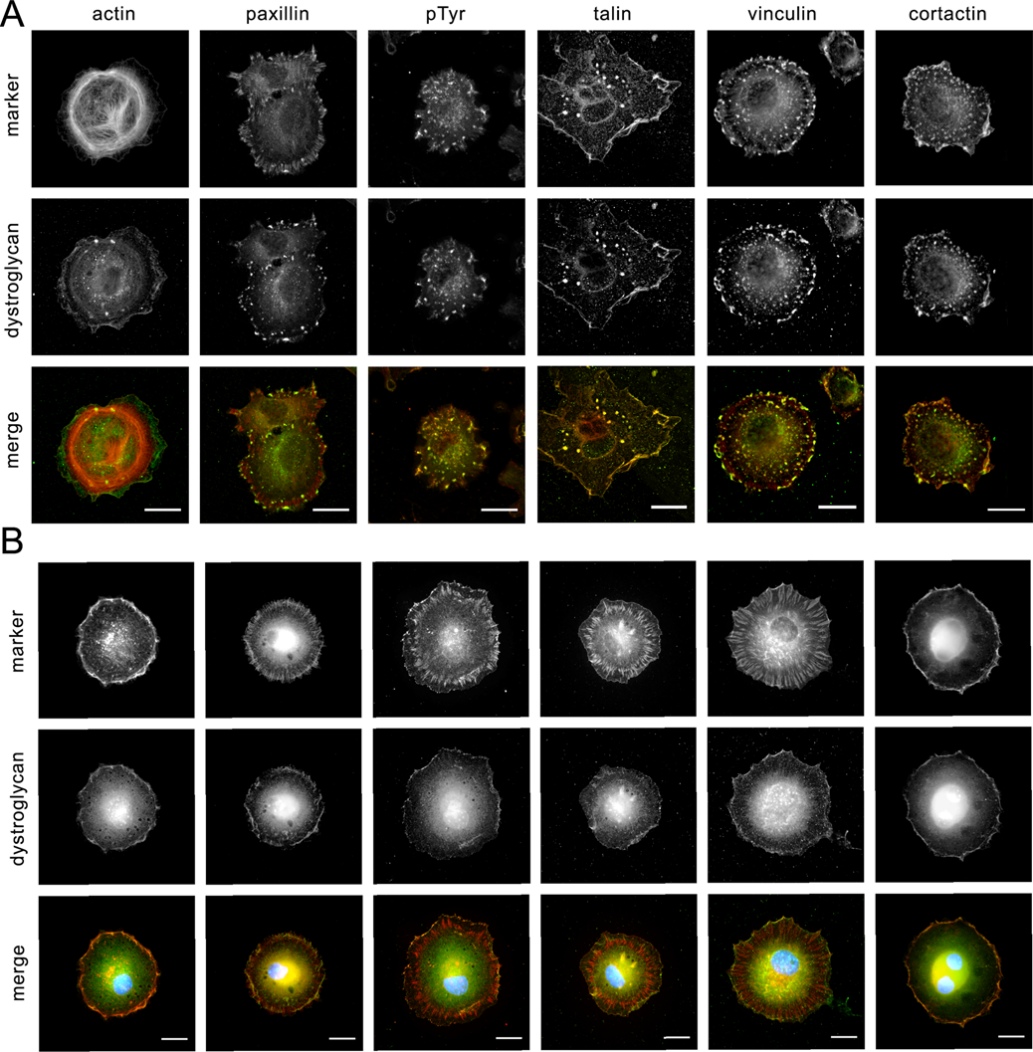
Src inhibition prevents dystroglycan localisation/podosome formation. Myoblast cells were seeded onto tissue culture plastic and allowed to spread for one hour before fixation and staining for β-dystroglycan, F-actin and the indicated markers of cell adhesion. In A cells were untreated and formed dystroglycan-containing puncta as before. Cells in B, were treated with the Src Inhibitor PP2 (10 µM) for 1 hour before and during re-seeding. No dystroglycan-containing puncta were formed in these cells. Focal adhesions appeared to form as normal, as judged by staining for paxillin, talin, vinculin and phospho-tyrosine (pTyr). Dystroglycan staining in green in all merged images. Scale bars 20 µm.

**Figure 9:**
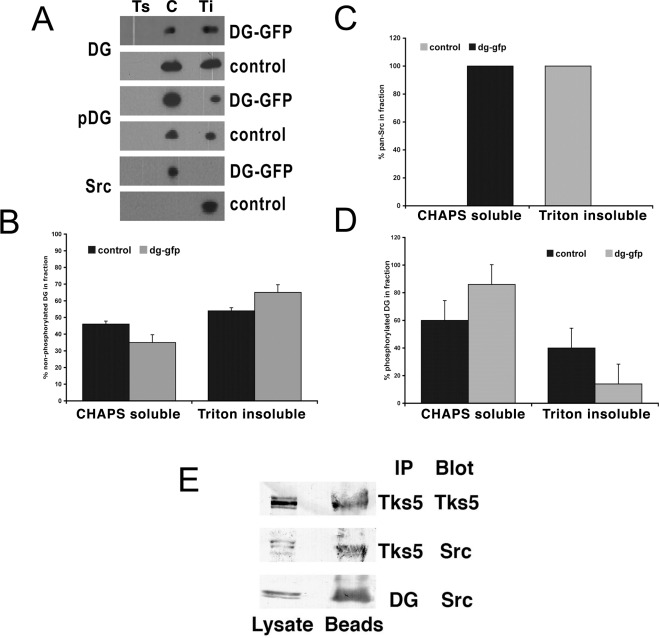
Dystroglycan overexpression alters Src distribution. A. A representative blot of fractionation of DG, pDG and Src in control or DG-GFP overexpressing cells; Ts, Triton soluble wash fraction; C, CHAPS soluble cytoplasmic/membrane fraction; Ti, Triton insoluble cytoskeletal fraction. Quantification from 3 independent fractionation experiments of DG (B), pDG (D) and pan Src (C) in control or DG-GFP overexpressing cells revealed an increase in pDG relative to total DG, and in the CGAPS soluble fraction relative to Triton insoluble cytoskeletal fraction, of DG overexpressing cells (B,D) Most dramatic and unexpected is a complete shift of Src from Triton insoluble cytoskeletal fraction to CHAPS soluble membrane fraction on DG overexpression (C). Immunoprecipitation from myoblast lysates with Tks5 or DG antibodies both revealed the presence of Src in the precipitated fraction of both suggesting the presence of a DG/Src/Tks5 complex in myoblast cells (E).

The dominant-negative effect of excess DG on podosome formation provided a useful tool to reveal functionally important regions of dystroglycan involved in podosome formation. By mutating potential binding sites or phosphorylation sites within the cytoplasmic domain of β-DG we could also potentially restore podosome formation by inhibiting the dominant-negative effect of DG overexpression. We therefore mutagenised potential tyrosine phosphorylation sites and proline residues in the SH3 domain binding consensus PxxP motifs within the β-DG cytoplasmic domain that might mediate SH3 domains interactions with Tks5. As can be seen in Figure 7, most mutations did not significantly reduce the inhibition of podosome formation compared to control, as dystroglycan-GFP carrying these mutations still had a dominant-negative effect. This was not due to the constructs failing to express as can be seen from the inset, the levels of DG-GFP, despite being a transient expression, are relatively similar. The fact these mutants did not behave exactly like wild-type and completely inhibit podosome formation, may reflect short range conformational effects induced by the mutations that perturbed interactions in neighbouring regions of the β-dystroglycan cytoplasmic domain. However mutations in Y890 restored podosome formation in myoblasts suppressing the dominant-negative effect of over-expressing dystroglycan-GFP (Figure 7). This result suggests that the phosphorylation of Y890 by Src is an important event in podosome formation. Overexpression of Y890A DG significantly restored podosomes to 70% of control levels, whereas mutation of other tyrosine residues or mutation of potential SH3 binding sites did not significantly affect podosome formation (Figure 7). These results highlight the importance of phosphorylation of β-DG Y890 in podosome assembly and corroborate the finding that DG associated with Tks5 is tyrosine phosphorylated (Figure 3B). Earlier work has shown dystroglycan Y890 to be a substrate for Src family kinases. We therefore treated early spreading myoblast cells with the Src inhibitor PP2 to observe the effect on podosome assembly. Compared to untreated cells, PP2 completely prevented the formation of the dystroglycan-rich puncta or podosomes instead DG was localised to the cell periphery along with actin and cortactin (Figure 8AB). As we have shown previously inhibition of Src does not affect focal adhesion formation per se [44], accordingly paxillin, talin and vinculin are all visible in peripheral focal adhesion-like structures (Figure 8AB), rather Src activity appears to be essential for focal adhesion turnover or disassembly [45]. By contrast in podosomes, Src activity appears to be required for the phosphorylation of Tks5 and β-DG in podosome *assembly*. Phosphorylation of DG Y890 would form a potential SH2 domain interaction site and DG phosphorylated at this site has been demonstrated previously to interact with Src itself [46]. We therefore carried out cell fractionation experiments in cells expressing different levels of dystroglycan and examined the distribution of dystroglycan and Src between cytoplasmic, membrane and cytoskeletal fractions. Due to its association with the actin cytoskeleton, DG is not readily extracted from membranes by Triton, but rather partitions with the insoluble cytoskeletal fraction [47]. We therefore utilised a protocol first extracting in CHAPS to release DG from the membrane, followed by a triton extraction step to precipitate the cytoskeleton and associated proteins in the triton insoluble fraction. This method has been employed with previous success to extract ERM family proteins and dystroglycan [48], [49]. Overexpression of DG results in increased levels of free DG in the CHAPS soluble fraction ([8]; Figure 9A–D). Furthermore, compared with the cytoskeleton associated (triton insoluble) dystroglycan a greater proportion of the CHAPS soluble DG is phosphorylated on Y890. In control cells the distribution of Src is almost exclusively in the triton insoluble fraction, but in cells overexpressing DG-GFP, Src localisation switches dramatically to the CHAPS soluble fraction. It would therefore appear that Y890 phosphorylation results in the creation of an SH2 domain binding site which also recruits Src to the CHAPS soluble fraction (Figure 9A,). This is supported by the finding that Src can be co-immunoprecipitated with both Tks5 and dystroglycan, suggesting the existence of a ternary complex between Tks5, Src and dystroglycan (Figure 9E). DG overexpression can therefore redirect the regulatory functions of both Tks5 and Src, the combined effects of which result in the inhibition of podosome formation. This adds further support to the idea that levels of dystroglycan must be finely balanced to maintain an appropriate level of scaffolding and signalling to regulate podosome formation.

## Discussion

Dystroglycan is a ubiquitously expressed laminin receptor having a key role in branching epithelial morphogenesis and the maintenance of membrane stability in mature skeletal muscle. But its role in early stages of muscle development or in early stages of myoblast adhesion, spreading and migration are not known. Dystroglycan is clearly present at the early stages of myoblast migration [1], but mice specifically lacking dystroglycan in myoblasts at these stages have not been developed due to the early embryonic lethality of DAG1 null embryos [50]. Furthermore, in chimaeric mice with skeletal muscle dystroglycan deficiency, or mice with a conditional knockout of dystroglycan in their skeletal muscles [51], [52], myoblast migration early in development would not be expected to be affected.

Our previous studies have highlighted the importance of adhesion mediated signalling leading to the phosphorylation of dystroglycan on a key regulatory tyrosine - Y890 in mouse (Y892 in human) [7], [20]. Tyrosine phosphorylation of dystroglycan, as is the case with many other adhesion receptors, is involved in regulating the connection to the cytoskeleton in this case via dystrophin or utrophin [7], [20] but not with some other binding partners such as caveolin [53], [54]. Furthermore, studies from the Lisanti group have demonstrated that Y890 is a substrate of Src family kinases and in addition phosphorylation of Y890 by Src provides an SH2 domain interaction site for Src itself and other tyrosine kinases or adaptor proteins including Fyn, Csk, Nck, and Shc [46]. Whilst previous studies have identified α-dystroglycan in focal adhesion and cell adhesion type structures [49], [55], and its intracellular linker utrophin in focal adhesions [56] there is little convincing evidence for β-dystroglycan in such structures even though it is presumed to be there to link between α-dystroglycan and utrophin. In this study we have identified α- and β-dystroglycan with utrophin in adhesion structures in spreading myoblasts. But these adhesion structures have a morphology more typical of podosomes rather than focal adhesions or focal contacts. The identification of the podosome regulatory protein Tks5 [21], [41] as binding partner for β-dystroglycan and the demonstration that the two proteins were co-localised in podosome like structures but not in focal adhesions, further substantiates the identity of the adhesion structures in myoblasts as podosomes.

Activation of Src downstream of PDBu-mediated activation of PKC leads to the phosphorylation of DG on Y890. Y890 is within a potential SH3 domain interaction consensus PYVP, and as such could be involved in a direct interaction with Tks5 through the 3^rd^ SH3 domain identified in the original proteomic screen. However as co-immunoprecipitation of Tks5 and β-DG revealed that β-DG was phosphorylated on Y890, it is likely that Tks5 interacts with a different SH3 interaction site in dystroglycan as tyrosine phosphorylation in this context would be likely to disrupt the SH3 domain interaction. Furthermore overexpression of dystroglycan was able to direct the localisation of Src away from the triton insoluble cytoskeletal fraction and into the CHAPS soluble membrane and cytoplasmic fraction. Thus we have demonstrated a binary complex between dystroglycan and the Tks5 SH3 domain *in vitro*, and by GST-affinity chromatography demonstrated that dystroglycan from cell extracts can also bind the Tks5 SH3 domain, but in the cellular context the dystroglycan recovered on these columns appears to be phosphorylated on Y890. If the association of dystroglycan and Tks5, whilst mediated at least in part by an SH3 interaction, is strengthened by phosphorylation on Y890, this would suggest the involvement of a third protein capable of interacting with Tks5 and the potential SH2 domain interaction site on dystroglycan generated by Y890 phosphorylation. Such a protein could be Src, as both Tks5 and dystroglycan are substrates for Src [21], [41], [46] and Src can bind to dystroglycan via an SH2 mediated interaction [46]. The redistribution of Src from triton insoluble to CHAPS soluble fraction on dystroglycan overexpression, is also in agreement with a Src-dystroglycan interaction. The distribution of Src between triton-soluble and triton insoluble fractions is dependent on a number of factors, including the cell type, the adhesion status of the cell, extracellular matrix ligands, presence or absence of serum or other factors that stimulate signalling cascades. Whilst often found associated with the triton soluble fraction [57], growth of cells on different extracellular matrix substrates can dramatically alter this distribution [58]. In fibronectin-stimulated NIH3T3 fibroblasts for example, active Src is almost exclusively recovered in the triton insoluble fraction [59].

Finally, Tks5, Src and dystroglycan were recovered by immunoprecipitation apparently as a ternary complex. Based on this notion, one could postulate a potential mechanism for the roles of Tks5 and DG in podosome assembly. Under normal circumstances Tks5 and β-DG are not associated and not present in podosomes or other adhesion structures. Following activation of Src, DG is phosphorylated on Y890 creating an SH2 domain interaction site leading to the recruitment of active Src to DG [46]. The dystroglycan-Src complex then also binds to Tks5 leading to its phosphorylation and the participation of the whole complex in the podosome assembly process. In the presence of excess dystroglycan, for example when DG is over-expressed, DG can still be phosphorylated by Src, but the excess DG in the cell effectively titrates out the Src signal with respect to Tks5 levels, thus reducing Tks5 phosphorylation and inhibiting podosome formation. A 50% reduction in dystroglycan has a moderate effect on podosome formation compared to overexpression, possibly because it allows some dystroglycan∶Src∶Tks5 complexes to form and these are sufficient to allow a limited number of podosomes to form. However too much dystroglycan is completely inhibitory due to the mislocalisation of Src likely reducing Tks5 phosphorylation removing a key stimulus to podosome formation [21]. Tks5 has also recently been shown to be involved in other ternary complexes that facilitate podosome formation. In Src-transformed NIH3T3 (NIH-Src) cells, Tks5 was found to be associated with the adaptor protein Grb2, and N-WASP a regulator of actin polymerisation [60]. In this system, Src expression drives the Grb2-Tks5 interaction which then associates with N-WASP via numerous SH3 mediated interactions targeting actin polymerisation to sites of podosome formation. This and our study would suggest that Tks5 is a multifunctional scaffold able to recruit other adaptors, signalling, regulatory and adhesion proteins such as dystroglycan to podosomes via its multiple SH3 domains.

Dystroglycan is therefore a new addition to the increasing number of podosome components, though rather than a spectator is appears to be a key player in the Src and Tks5-dependent regulation of podosome formation in myoblasts. Moreover the demonstration of podosome formation in myoblasts opens new avenues to dissect cell migration during early stages of development and in muscle repair, where myoblast cells and their precursors travel large distances to populate newly developing muscles or to mend damaged or diseased muscle.

## Supporting Information

Figure S1 Localisation of dystroglycan and the indicated core podosome proteins in H2k myoblasts (A), C2C12 myoblasts (B) and A7r5 smooth muscle cells (C). Cells were stimulated to form podosomes with PDBu and stained for the indicated adhesion proteins or F-actin. In merged images dystroglycan is always green and/or F-actin is always red.
(10.06 MB DOC)

Movie S1 H2k myoblast transiently expressing actin-GFP and stimulated with PDBu. Frame interval in is 30 sec. Cell exhibits dense but transient actin puncta - podosomes at the cell periphery or more centrally beneath the nucleus in reponse to PDBu stimulation.
(0.72 MB MOV)

Movie S2 H2k myoblast transiently expressing actin-GFP and stimulated with PDBu. Frame interval in is 30 sec. Cell exhibits dense but transient actin puncta - podosomes at the cell periphery or more centrally beneath the nucleus in reponse to PDBu stimulation.
(1.24 MB MPG)

Movie S3 H2k myoblast transiently expressing actin-GFP and stimulated with PDBu. Frame interval in is 30 sec. Cell exhibits dense but transient actin puncta - podosomes at the cell periphery or more centrally beneath the nucleus in reponse to PDBu stimulation.
(1.41 MB MPG)

## References

[1] Anderson C, Winder SJ, Borycki A-G (2007). Dystroglycan protein distribution coincides with basement membranes and muscle differentiation during mouse embryogenesis.. Dev Dyn.

[2] Linder S (2007). The matrix corroded: podosomes and invadopodia in extracellular matrix degradation.. Trends in Cell Biology.

[3] Tarone G, Cirillo D, Giancotti FG, Comoglio PM, Marchisio PC (1985). Rous sarcoma virus-transformed fibroblasts adhere primarily at discrete protrusions of the ventral membrane called podosomes.. Exp Cell Res.

[4] Zhang D, Udagawa N, Nakamura I, Murakami H, Saito S (1995). The small GTP-binding protein, rho p21, is involved in bone resorption by regulating cytoskeletal organization in osteoclasts.. J Cell Sci.

[5] Linder S, Nelson D, Weiss M, Aepfelbacher M (1999). Wiskott-Aldrich syndrome protein regulates podosomes in primary human macrophages.. Proc Nat Acad Sci US.

[6] Gimona M, Buccione R (2006). Adhesions that mediate invasion.. Int J Biochem Cell Biol.

[7] James M, Nuttall A, Ilsley JL, Ottersbach K, Tinsley JN (2000). Adhesion-dependent tyrosine phosphorylation of β-dystroglycan regulates its interaction with utrophin.. J Cell Sci.

[8] Batchelor CL, Higginson JR, Chen Y-J, Vanni C, Eva A (2007). Recruitment of Dbl by ezrin and dystroglycan drives membrane proximal Cdc42 activation and filopodia formation.. Cell Cycle.

[9] Smalheiser NR, Schwartz NB (1987). Cranin a laminin-binding protein of cell membranes.. Proc Natl Acad Sci USA.

[10] Ibraghimov-Beskrovnaya O, Ervasti JM, Leveille CJ, Slaughter CA, Sernett SW (1992). Primary structure of dystrophin-associated glycoproteins linking dystrophin to the extracellular matrix.. Nature.

[11] Winder SJ (2001). The complexities of dystroglycan.. Trends Biochem Sci.

[12] Higginson JR, Thompson O, Winder SJ (2008). Targeting of dystroglycan to the cleavage furrow and midbody in cytokinesis.. Int J Biochem Cell Biol.

[13] Ervasti JM, Ohlendieck K, Kahl SD, Gaver MG, Campbell KP (1990). Deficiency of a glycoprotein component of the dystrophin complex in dystrophic muscle.. Nature.

[14] Batchelor C, Winder S (2006). Sparks, signals and shock absorbers: how dystrophin loss causes muscular dystrophy.. Trends Cell Biol.

[15] Thompson O, Higginson JR, Batchelor CL, Spence HJ, Chen Y-J, Fattoum A (2007). Dystroglycan: a multifunctional adaptor protein.. Gafsa- A Focus on Biochemistry and Genetics: from Concepts to Therapeutic Advances: Repro-France Industries.

[16] Morgan JE, Beauchamp JR, Pagel CN, Peckham M, Ataliotis P (1994). Myogenic Cell Lines Derived from Transgenic Mice Carrying a Thermolabile T Antigen: A Model System for the Derivation of Tissue-Specific and Mutation-Specific Cell Lines.. Dev Biol.

[17] Wizemann H, Garbe JH, Friedrich MV, Timpl R, Sasaki T (2003). Distinct requirements for heparin and alpha-dystroglycan binding revealed by structure-based mutagenesis of the laminin alpha2 LG4-LG5 domain pair.. J Mol Biol.

[18] Spence HJ, Chen Y-J, Batchelor CL, Higginson JR, Suila H (2004). Ezrin-dependent regulation of the actin cytoskeleton by β-dystroglycan.. Hum Mol Genet.

[19] Pereboev A, Ahmed N, Man Nt, Morris G (2001). Epitopes in the interacting regions of beta-dystroglycan (PPxY motif) and dystrophin (WW domain).. Biochim Biophys Acta.

[20] Ilsley JL, Sudol M, Winder SJ (2001). The interaction of dystrophin with β-dystroglycan is regulated by tyrosine phosphorylation.. Cell Signal.

[21] Seals DF, Azucena JEF, Pass I, Tesfay L, Gordon R (2005). The adaptor protein Tks5/Fish is required for podosome formation and function, and for the protease-driven invasion of cancer cells.. Cancer Cell.

[22] Zicha D, Dobbie IM, Holt MR, Monypenny J, Soong DYH (2003). Rapid Actin Transport During Cell Protrusion.. Science.

[23] Chen Y-J, Spence HJ, Cameron JM, Jess T, Ilsley JL (2003). Direct interaction of β-dystroglycan with F-actin.. Biochem J.

[24] Kärkkäinen S, Hiipakka M, Wang J, Kleino I, Vähä-Jaakkola M (2006). Identification of preferred protein interactions via phage-display of the human Src homology-3 proteome.. EMBO Rep.

[25] Pistor S, Chakraborty T, Niebuhr K, Domann E, Wehland J (1994). The ActA protein of Listeria monocytogenes acts as a nucleator inducing reorganization of the actin cytoskeleton.. EMBO J.

[26] Grubinger M, Gimona M (2004). CRP2 is an autonomous actin-binding protein.. FEBS Letters.

[27] Pertz O, Hodgson L, Klemke RL, Hahn KM (2006). Spatiotemporal dynamics of RhoA activity in migrating cells.. Nature.

[28] Ervasti JM (2003). Costameres: the Achilles' Heel of Herculean Muscle.. J Biol Chem.

[29] Nobes CD, Hall A (1995). Rho, Rac, and cdc42 GTPases Regulate the Assembly of Multimolecular Focal Complexes Associated with Actin Stress Fibers, Lamellipodia, and Filopodia.. Cell.

[30] Hotchin NA, Hall A (1995). The assembly of integrin adhesion complexes requires both extracellular matrix and intracellular rho/rac GTPases.. J Cell Biol.

[31] Tatin F, Varon C, Genot E, Moreau V (2006). A signalling cascade involving PKC, Src and Cdc42 regulates podosome assembly in cultured endothelial cells in response to phorbol ester.. J Cell Sci.

[32] Gad A, Lach S, Crimaldi L, Gimona M (2008). Plectin deposition at podosome rings requires myosin contractility.. Cell Motil Cytoskel in press: 1097-0169 1010.1002/cm.20287.

[33] Bains W, Ponte P, Blau H, Kedes L (1984). Cardiac actin is the major actin gene product in skeletal muscle cell differentiation in vitro.. Mol Cell Biol.

[34] Webb BA, Eves R, Crawley SW, Zhou S, Cote GP (2005). PAK1 induces podosome formation in A7r5 vascular smooth muscle cells in a PAK-interacting exchange factor-dependent manner.. Am J Physiol Cell Physiol.

[35] Burgstaller G, Gimona M (2004). Actin cytoskeleton remodelling via local inhibition of contractility at discrete microdomains.. J Cell Sci.

[36] Kaverina I, Stradal TEB, Gimona M (2003). Podosome formation in cultured A7r5 vascular smooth muscle cells requires Arp2/3-dependent de-novo actin polymerization at discrete microdomains.. J Cell Sci.

[37] Zaidel-Bar R, Itzkovitz S, Ma'ayan A, Iyengar R, Geiger B (2007). Functional atlas of the integrin adhesome.. Nat Cell Biol.

[38] Lock P, Abram CL, Gibson T, Courtneidge SA (1998). A new method for isolating tyrosine kinase substrates used to identify fish, an SH3 and PX domain-containing protein, and Src substrate.. EMBO J.

[39] Yang B, Jung D, Motto D, Meyer J, Koretzky G (1995). SH3 domain-mediated interaction of dystroglycan and Grb2.. J Biol Chem.

[40] Russo K, Di Stasio E, Macchia G, Rosa G, Brancaccio A (2000). Characterization of the beta-dystroglycan-growth factor receptor 2 (Grb2) interaction.. Biochem Biophys Res Commun.

[41] Abram CL, Seals DF, Pass I, Salinsky D, Maurer L (2003). The Adaptor Protein Fish Associates with Members of the ADAMs Family and Localizes to Podosomes of Src-transformed Cells.. J Biol Chem.

[42] Blouw B, Seals DF, Pass I, Diaz B, Courtneidge SA (2008). A role for the podosome/invadopodia scaffold protein Tks5 in tumor growth in vivo.. Eur J Cell Biol.

[43] Evans JG, Correia I, Krasavina O, Watson N, Matsudaira P (2003). Macrophage podosomes assemble at the leading lamella by growth and fragmentation.. J Cell Biol.

[44] Fincham VJ, James M, Frame MC, Winder SJ (2000). Active ERK/MAP kinase is targeted to newly forming cell-matrix adhesions by integrin engagement and v-Src.. EMBO J.

[45] Fincham VJ, Frame MC (1998). The catalytic activity of Src is dispensable for translocation to focal adhesions but controls the turnover of these structures during cell motility.. EMBO J.

[46] Sotgia F, Lee H, Bedford M, Petrucci TC, Sudol M (2001). Tyrosine phosphorylation of b-dystroglycan at its WW domain binding motif, PPxY, recruits SH2 domain containing proteins.. Biochemistry.

[47] Campbell KP, Kahl SD (1989). Association of dystrophin and an integral membrane glycoprotein.. Nature.

[48] Lamb RF, Ozanne BW, Roy C, McGarry L, Stipp C (1997). Essential functions of ezrin in maintenance of cell shape and lamellipodial extension in normal and transformed fibroblasts.. Curr Biol.

[49] Spence HJ, Dhillon AS, James M, Winder SJ (2004). Dystroglycan a scaffold for the ERK-MAP kinase cascade.. EMBO Rep.

[50] Williamson RA, Henry MD, Daniels KJ, Hrstka RF, Lee JC (1997). Dystroglycan is essential for early embryonic development: disruption of Reichert's membrane in *Dag1*-null mice.. Hum Mol Genet.

[51] Cohn RD, Henry MD, Michele DE, Barresi R, Saito F (2002). Disruption of DAG1 in differentiated skeletal muscle reveals a role for dystroglycan in muscle regeneration.. Cell.

[52] Cote PD, Moukhles H, Lindenbaum M, Carbonetto S (1999). Chimeric mice deficient in dystroglycans develop muscular dystrophy and have disrupted myoneural synapses.. Nature Genet.

[53] Ilsley JL, Sudol M, Winder SJ (2002). The WW domain: linking cell signalling to the membrane cytoskeleton.. Cell Signal.

[54] Sotgia F, Lee JK, Das K, Bedford M, Petrucci TC (2000). Caveolin-3 directly interacts with the c-terminal tail of β-dystroglycan: identification of a central WW-like domain within caveolin family members.. J Biol Chem.

[55] Belkin AM, Smalheiser NR (1996). Localization of cranin (dystroglycan) at sites of call-matrix and cell-cell contact: recruitment to focal adhesions is dependent upon extracellular ligands.. Cell Adhesion Commun.

[56] Belkin AM, Burridge K (1995). Localization of utrophin and aciculin at sites of cell-matrix and cell-cell adhesion in cultured cells.. Exp Cell Res.

[57] Kaplan KB, Bibbins KB, Swedlow JR, Arnaud M, Morgan DO (1994). Association of the amino-terminal half of c-Src with focal adhesions alters their properties and is regulated by phosphorylation of tyrosine 527.. EMBO J.

[58] Kaplan KB, Swedlow JR, Morgan DO, Varmus HE (1995). c-Src enhances the spreading of src−/− fibroblasts on fibronectin by a kinase-independent mechanism.. Genes Dev %R 101101/gad9121505.

[59] Schlaepfer DD, Jones KC, Hunter T (1998). Multiple Grb2-Mediated Integrin-Stimulated Signaling Pathways to ERK2/Mitogen-Activated Protein Kinase: Summation of Both c-Src- and Focal Adhesion Kinase-Initiated Tyrosine Phosphorylation Events.. Mol Cell Biol.

[60] Oikawa T, Itoh T, Takenawa T (2008). Sequential signals toward podosome formation in NIH-src cells.. J Cell Biol.

