# Supplementary material for: Dystroglycan, Tks5 and Src Mediated Assembly of Podosomes in Myoblasts
Source: PLoS One. 2008 Nov 4;3(11):e3638. doi: 10.1371/journal.pone.0003638 (PMC2572840; doi:10.1371/journal.pone.0003638)
Supplement: Figure S1 — Localisation of dystroglycan and the indicated core podosome proteins in H2k myoblasts (A), C2C12 myoblasts (B) and A7r5 smooth muscle cells (C). Cells were stimulated to form podosomes with PDBu and stained for the indicated adhesion proteins or F-actin. In merged images dystroglycan is always green and/or F-actin is always red. (10.06 MB DOC) [file pone.0003638.s001.doc]

**Figure S1A**. Other proteins in podosomes in H2k myoblasts


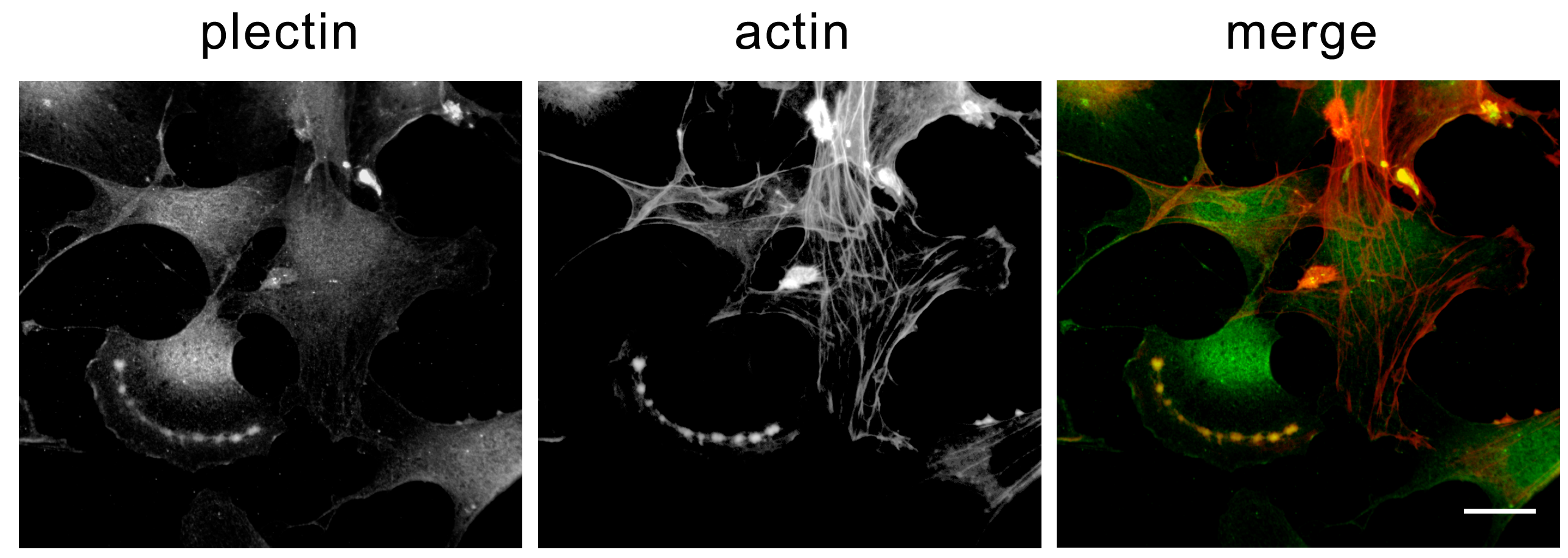

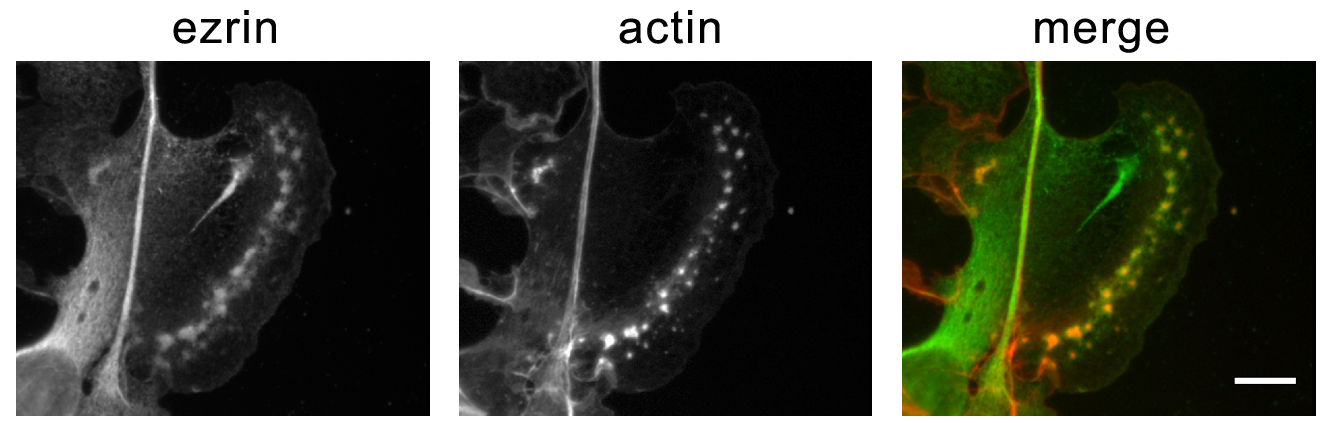


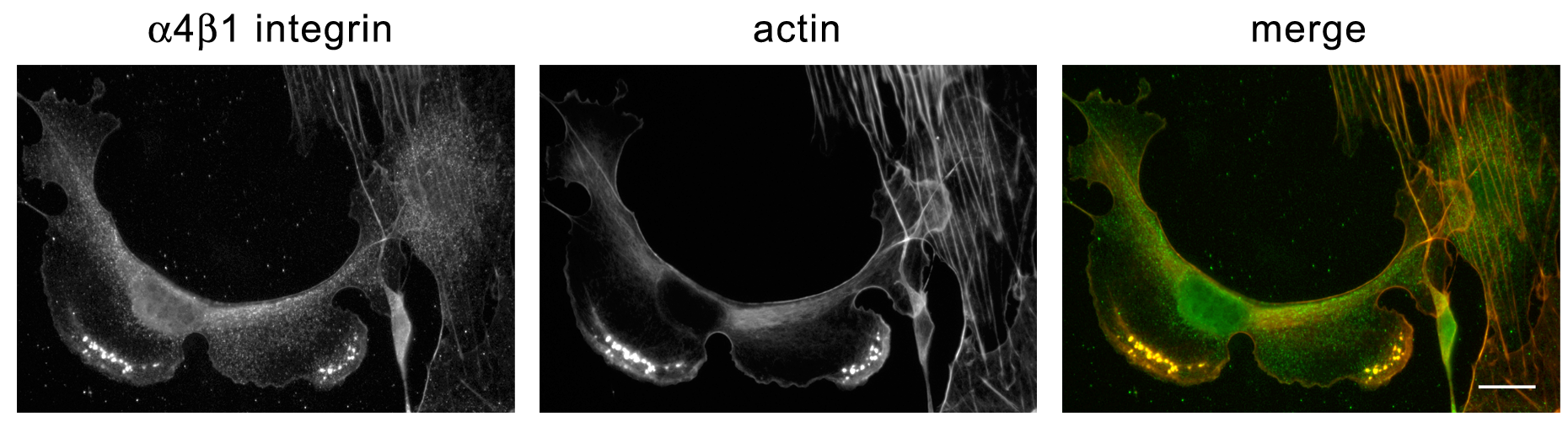


**Figure S1B** Formation of podosomes in C2C12 cells


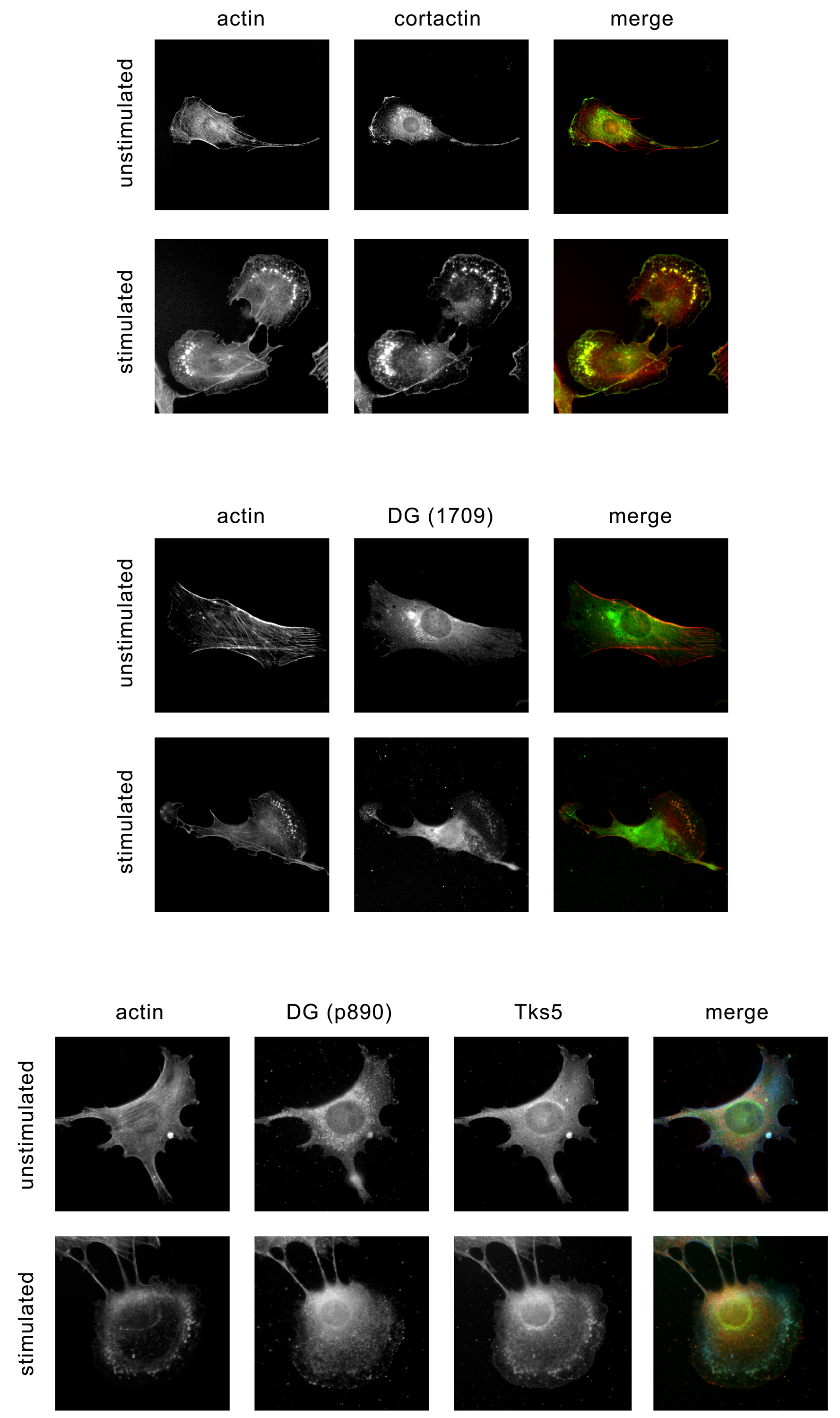


**Figure S1C**. Dystroglycan and core podosome proteins in A7r5 cells


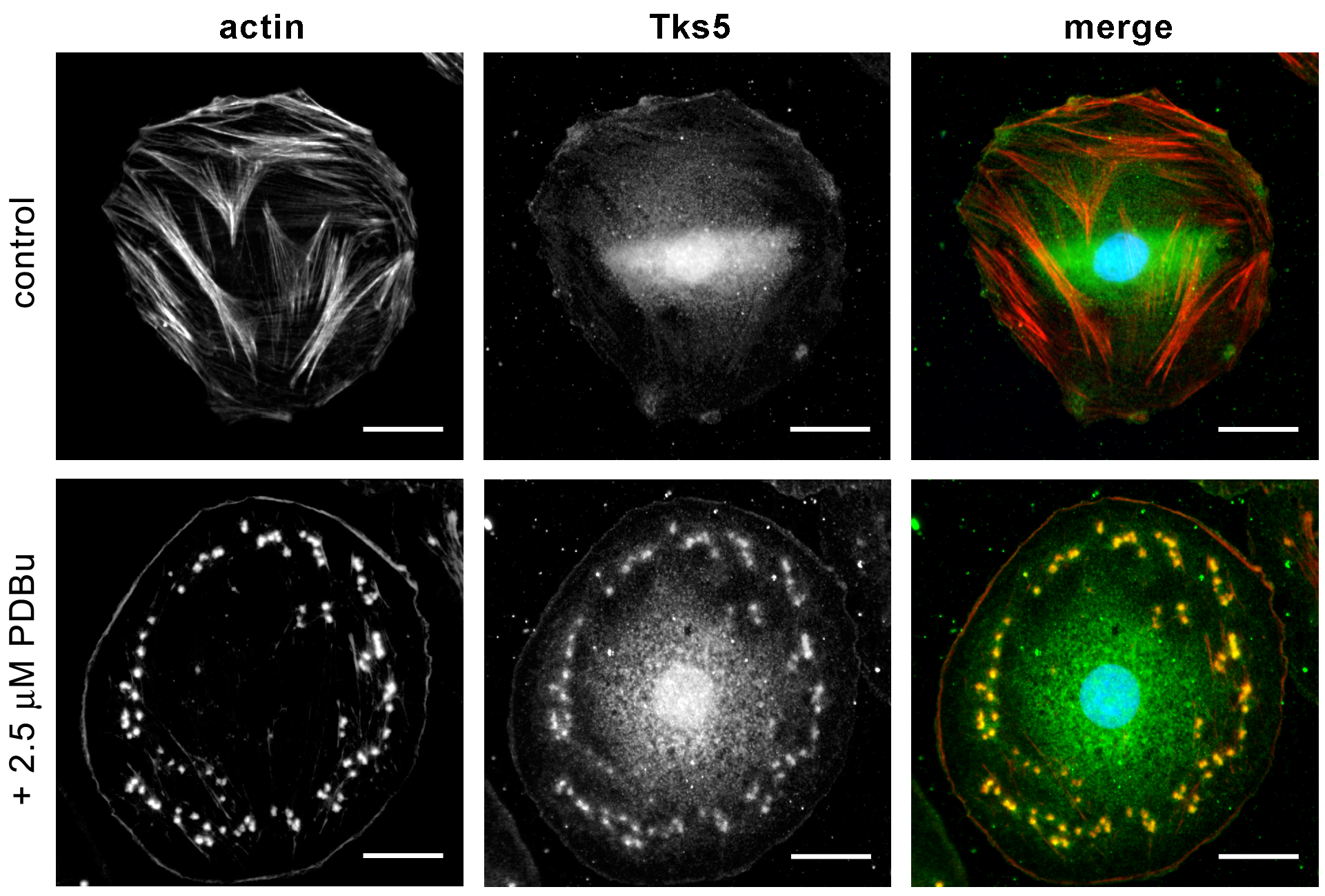


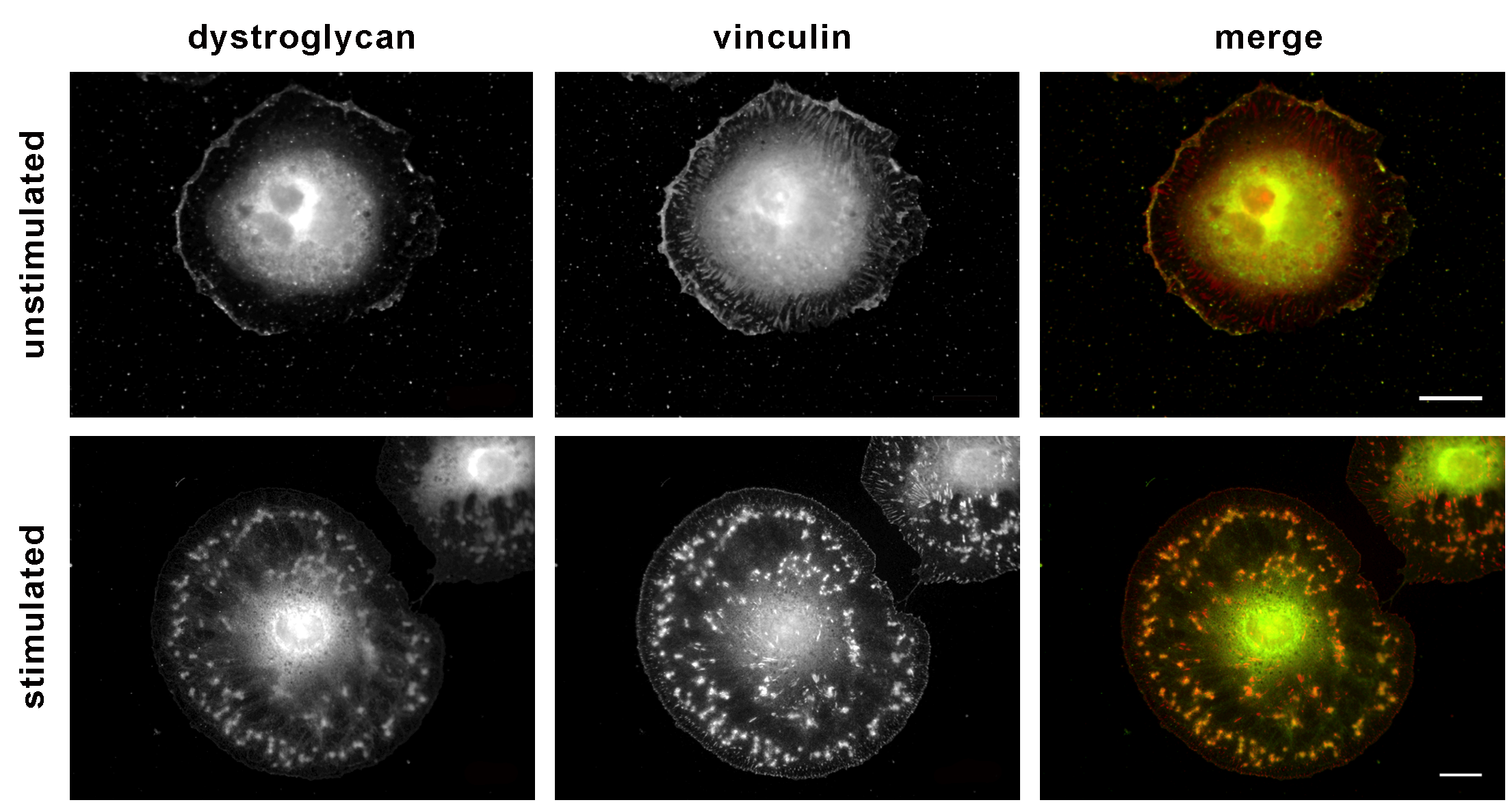


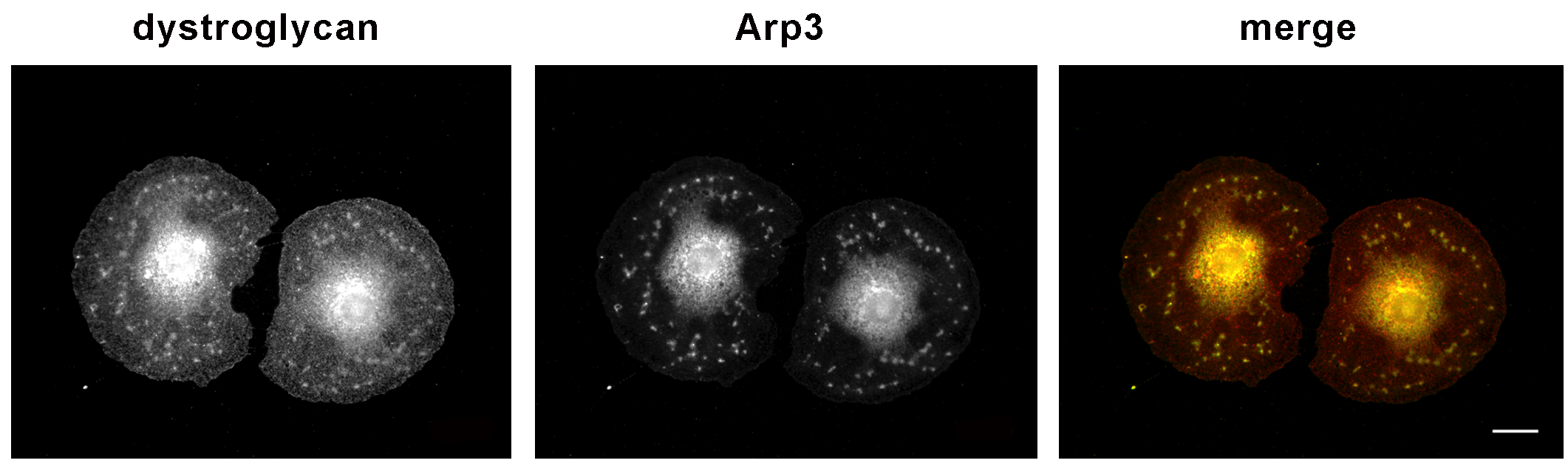


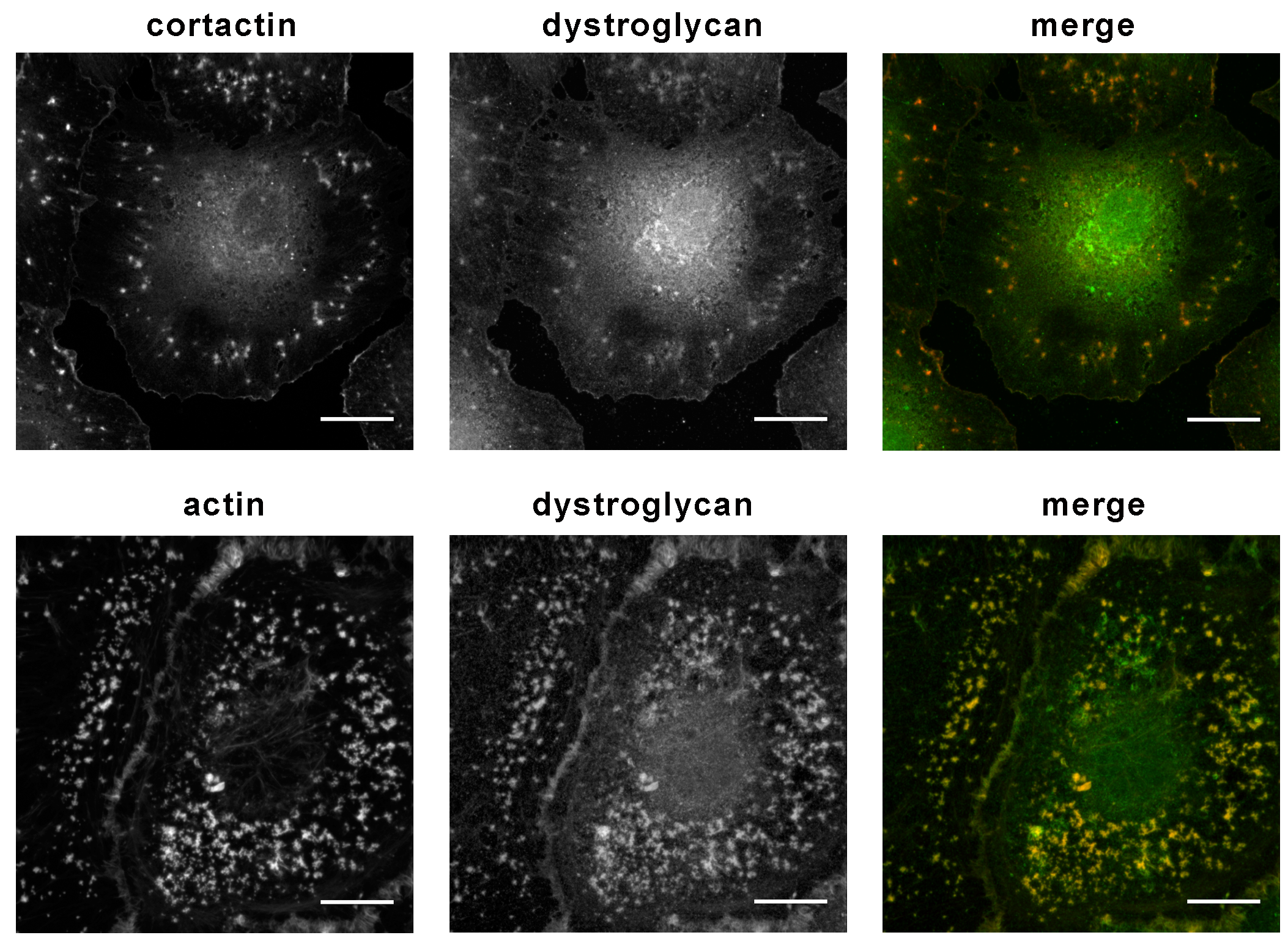


Figure S1. Localisation of dystroglycan and the indicated core podosome proteins in H2k myoblasts (A), C2C12 myoblasts (B) and A7r5 smooth muscle cells (C). Cells were stimulated to form podosomes with PDBu and stained for the indicated adhesion proteins or F-actin. In merged images dystroglycan is always green and/or F-actin is always red.
